# Molecular and neural mechanisms of sex pheromone reception and processing in the silkmoth *Bombyx mori*

**DOI:** 10.3389/fphys.2014.00125

**Published:** 2014-03-31

**Authors:** Takeshi Sakurai, Shigehiro Namiki, Ryohei Kanzaki

**Affiliations:** Intelligent Cooperative Systems, Research Center for Advanced Science and Technology, The University of TokyoMeguro-ku, Japan

**Keywords:** insect, silkmoth, olfaction, sex pheromone, pheromone-source searching behavior

## Abstract

Male moths locate their mates using species-specific sex pheromones emitted by conspecific females. One striking feature of sex pheromone recognition in males is the high degree of specificity and sensitivity at all levels, from the primary sensory processes to behavior. The silkmoth *Bombyx mori* is an excellent model insect in which to decipher the underlying mechanisms of sex pheromone recognition due to its simple sex pheromone communication system, where a single pheromone component, bombykol, elicits the full sexual behavior of male moths. Various technical advancements that cover all levels of analysis from molecular to behavioral also allow the systematic analysis of pheromone recognition mechanisms. Sex pheromone signals are detected by pheromone receptors expressed in olfactory receptor neurons in the pheromone-sensitive sensilla trichodea on male antennae. The signals are transmitted to the first olfactory processing center, the antennal lobe (AL), and then are processed further in the higher centers (mushroom body and lateral protocerebrum) to elicit orientation behavior toward females. In recent years, significant progress has been made elucidating the molecular mechanisms underlying the detection of sex pheromones. In addition, extensive studies of the AL and higher centers have provided insights into the neural basis of pheromone processing in the silkmoth brain. This review describes these latest advances, and discusses what these advances have revealed about the mechanisms underlying the specific and sensitive recognition of sex pheromones in the silkmoth.

## Introduction

Olfactory information plays pivotal roles in many aspects of an animal's life including foraging, prey detection, finding hosts, and mating. Animals can extract adequate information from the numerous odorants in their surroundings to respond in the appropriate behavioral manner. Clarification of the mechanisms by which animals detect olfactory information, process it in the brain, and finally translate it into the appropriate behavioral responses is of critical importance in neuroscience.

The insect brain provides an excellent model system for deciphering the neural mechanisms underlying olfactory-driven behavior for two reasons. For one, it consists of far fewer neurons (10^5^–10^6^) than the mammalian brain, which allows the examination of the whole brain as a system (Mizunami et al., 2004). In addition, despite of such small brain, insects exhibit stereotypic innate behaviors in response to specific environmental stimuli such as sex pheromones and CO_2_ showing robust and relatively straightforward input-output relationships. The sex pheromone and its associated pheromone-source searching behavior is one of the best examples of these relationships. In particular, the silkmoth *Bombyx mori* has the simplest possible sex pheromone system, in which a single pheromone compound releases programmed pheromone-source searching behavior, while most moth species utilize more complex system where blends of several pheromone components are required for behavioral responses in males. Female silkmoths produce two pheromone components: bombykol [10,12-(*E*,*Z*)-hexadecadien-1-ol] and bombykal [10,12-(*E*,*Z*)-hexadecadien-1-al] at about 10:1 ratio in pheromone glands (Butenandt et al., 1959; Kaissling et al., 1978; Schneider, 1992); however, bombykol alone is sufficient to trigger pheromone-source searching behavior in male moths (Kramer, 1975; Kaissling, 1987). Though we refer to bombykal as a minor pheromone component in the present review according to the convention, biological activity of bombykal seems to be unusual for sex pheromone components in moths; bombykal is supposed not to contribute to attraction of male moths but to suppress initiation of the pheromone-source searching behavior triggered by bombykol (Kaissling et al., 1978; Daimon et al., 2012b). Further, some *B. mori* strains do not produce bombykal (Daimon et al., 2012b). Therefore, further investigation may be necessary to define bombykal as a sex pheromone component of *B. mori*. Regardless of such a unique effect of bombykal, a straightforward input-output relationship between bombykol and pheromone-source searching behavior allows researchers to easily correlate molecular and neural functions with behavioral responses. Pheromone-source searching behavior can also be modulated by several internal and external factors, including non-bombykol odorant stimuli (Namiki et al., 2008), circadian rhythms, and associated serotonin levels in the brain (Gatellier et al., 2004). Therefore, *B. mori* is also a good model in which to study the neural mechanisms by which programmed behavior is modulated dynamically in response to external stimuli and changes in the internal state.

In addition to the biological features of *B. mori*, various experimental techniques that enable the examination of the mechanisms at all levels including a single neuron, a neuronal network, and behavior are well-established in *B. mori* (Kanzaki et al., 2008). Recent clarification of the whole genome sequence (The International Silkworm Genome Consortium, 2008) and the availability of various genetic manipulation techniques including the use of transgenes (Tamura et al., 2000; Imamura et al., 2003) and gene-targeting (Daimon et al., 2013) make *B. mori* an excellent model system for studying the genetic basis underlying pheromone detection and processing in relation to the function of neural circuits.

In this review, we summarize the current knowledge on the olfactory circuits of *B*. *mori*. There are multiple steps involved in the reception and processing of sex pheromone signals into behavioral responses. We trace the path of information flow from the reception of sex pheromone molecules in the air by the olfactory organ to processing in the brain and the generation of the neural activity patterns responsible for the programmed behavioral responses.

## Structure of antennae

**Figure 1:**
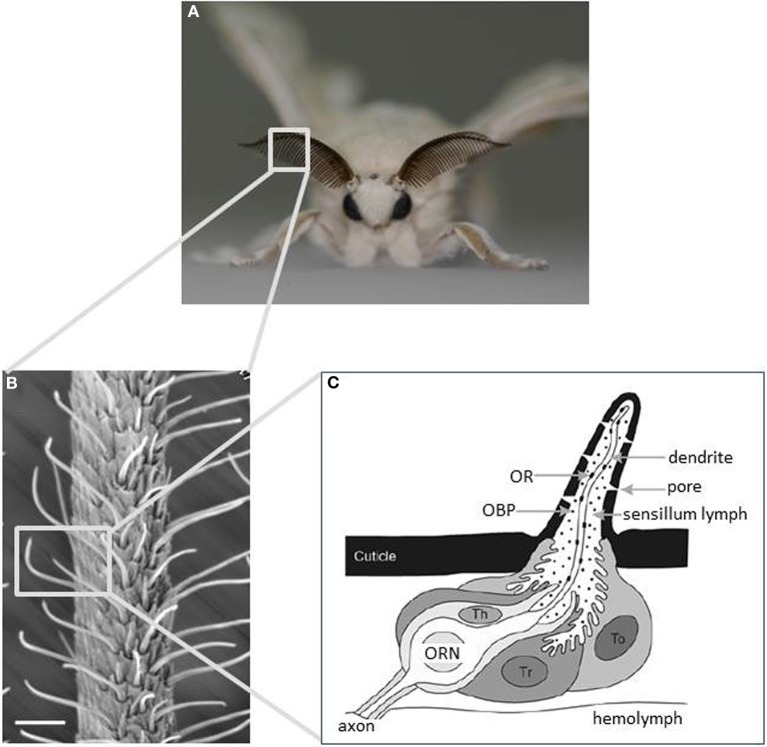
**Main olfactory sensory organs of the silkmoth *Bombyx mori*. (A)** A male silkmoth with its prominent antennae optimized for odorant detection. **(B)** Scanning electron micrograph of an antenna displaying the external morphology of sensilla trichodea. Scale bar: 25 μm. **(C)** Schematic diagram of an olfactory sensillum showing the detailed configuration of ORNs and accessory cells with respect to cuticular specializations. Three types of accessory cell surround the cell bodies of ORNs: tormogen (To), trichogen (Tr), and thecogen cells (Th). To and Tr cells secrete odorant-binding proteins into the sensillum lymph. Odorants are detected by ORs expressed on the dendritic membrane of ORNs. (modified from Jacquin-Joly and Merlin, 2004).

Moths detect odorants using a pair of antennae on their head. The structure of moth antennae, particularly in males, is often optimized to detect odorants. The silkmoth has a bipectinate antenna formed by pairs of antennal side branches that stem from the antennal stalk (Figure 1A). Olfactory receptor neurons (ORNs) are housed in a cuticular specialization called the olfactory sensillum on the antennae. Their dendrites extend into the sensillum and their axons project into the first olfactory center of the brain, the antennal lobe (AL). Most olfactory sensilla are arranged in a regular array on the inner side of the antennal branches (Figure 1B) (Steinbrecht, 1970). This configuration forms a molecular sieve to efficiently catch pheromone molecules passing over the antenna (Koehl, 2005). On the male antenna, there are ~25,000 olfactory sensilla that are categorized into at least four morphological types: long trichodea, medium trichodea, basiconica, and coeloconica. Most (~75%) are long trichodea sensilla (Steinbrecht, 1970) that house a pair of ORNs tuned specifically to detect bombykol and bombykal (Kaissling et al., 1978). Other types house ORNs tuned to general odorants such as plant-derived odorants (Pophof, 1997).

Calculations based on electrophysiological studies using radiolabeled bombykol have revealed that bombykol-sensitive ORNs are so sensitive that a single pheromone molecule can evoke an electrical signal (Kaissling, 1987). In addition, these receptor neurons show extremely high selectivity, and generally respond only to a single pheromone component (Kikuchi, 1975). In the following sections, we summarize and discuss the recent evidence of the underlying molecular mechanisms for the specificity and sensitivity of pheromone reception in *B. mori*.

## Molecular components of pheromone reception

### Overview of pheromone reception

Pheromone molecules are first absorbed by the cuticular surface of a sensillum, and then diffuse inside through olfactory pores and the pore tubule (Kasang and Kaissling, 1972; Kanaujia and Kaissling, 1985; Kaissling, 1987). Because volatile pheromones are highly hydrophobic, they do not pass easily across the sensillum lymph surrounding the dendritic membrane of pheromone-sensitive ORNs. However, by binding to pheromone-binding proteins (PBPs) in the sensillum lymph, they become solubilized and then pass more easily. Sex pheromone receptor proteins expressed on the dendritic membrane, which trigger the activation of the chemo-electrical transduction machinery, recognize pheromones delivered to, and released in the vicinity of, the dendritic membrane of pheromone receptor neurons. After activating pheromone receptors, the pheromone molecules are inactivated either by enzymatic degradation or via unidentified rapid inactivation mechanisms that may occur before enzymatic degradation.

### Pheromone-binding protein

The PBP family is a subfamily of the odorant-binding protein (OBP) family in insects, which were defined originally by their ability to bind to pheromone compounds and by showing a predominant expression pattern in male antennae in the giant silkmoth *Antheraea polyphemus* (Vogt and Riddiford, 1981). PBPs are small, soluble proteins that are synthesized by two out of three accessory cells (trichogen, or tormogen cells), and are secreted abundantly into the sensillum lymph at concentrations of up to 10 mM (Klein, 1987). Because the sensillum lymph is isolated between each sensillum by accessory cells, ORNs in different sensilla can be surrounded by lymph fluid containing different molecular constituents (Figure 1C). In *B. mori*, the expression of OBPs is correlated with the morphological type of sensillum: the lymph of the pheromone-sensitive long sensilla trichodea (s. trichodea) contains BmPBP1, whereas other sensillum types and long s. trichodea in females that are tuned to general odorants express other OBPs (Steinbrecht et al., 1995; Maida et al., 2005). In addition to BmPBP1, two PBP-like genes, BmPBP2 and BmPBP3, have been reported (Forstner et al., 2006), along with 41 OBPs in the silkmoth genome (Gong et al., 2009). Transcripts of these PBP genes are detected in cells that do not overlap with BmPBP1 cells, suggesting that BmPBP2 and 3 function in sensilla other than long s. trichodea.

The mechanisms of pheromone binding and release of BmPBP1 are some of the best-characterized biochemical and structural examples of OBP functioning. BmPBP1 undergoes a conformational change when pH becomes more acidic, as is predicted at the proximity of a cell membrane, which could result in the release of bound pheromones onto the dendritic membrane of the ORN (Wojtasek and Leal, 1999). This hypothesis is supported by structural analyses, which have revealed that conformational changes at acidic pH lead to the release of bound bombykol from BmPBP1 (Tegoni et al., 2004). First, X-ray diffraction analysis of a BmPBP1 crystal complexed with bombykol that was grown at neutral pH revealed that BmPBP1 consists of 6 α-helices. Of these, 4 antiparallel helices form the binding pocket that completely encloses a bombykol molecule in the core of protein (Sandler et al., 2000). Then, the NMR structure of the unliganded BmPBP1 was determined at acidic (pH 4.5) (Horst et al., 2001) and neutral pH (pH 6.5) (Lee et al., 2002). The most striking structural differences are found at C-terminal ends. At acidic pH, the C-terminal dodecapeptide forms a 7th helix that occupies the binding pocket for bombykol, while C-terminal end is present as a flexible loop structure at neutral pH. This conformational transition was suggested to be the mechanisms for the release of the ligand at the proximity of a cell membrane.

Interestingly, this pH-dependent conformational transition of BmPBP1 is affected by the presence or absence of ligands, and their structures (Damberger et al., 2013). The transition midpoint pH in the presence of bombykol (~pH 5.37) is much lower than in the absence of ligands (~pH 7.25) and slightly lower than in the presence of structurally related ligands (~pH 5.75), suggesting that the affinity of BmPBP1 is higher for bombykol than related ligands at low pH binding. Based on the pH- and ligand-dependent conformational transition, the following model for the selective transport of bombykol to the sex pheromone receptor has been proposed: (1) BmPBP1 preferentially uptakes bombykol at areas of reduced pH near the olfactory pore; (2) during transport, bombykol is protected from degradation by odorant degrading enzyme (ODE) in sensillum lymph by binding to the cavity of BmPBP1; (3) the acidic pH around the membrane allows the release of bombykol from BmPBP1, resulting in its reception by the sex pheromone receptor protein BmOR1. Meanwhile, because the binding affinity of related ligands is lower than that of bombykol, they are released before they reach the vicinity of the membrane, and are supposed to be degraded by ODE (Damberger et al., 2013). This study showed that not only the binding specificity of PBP but also its binding dynamics may play a role in enhancing the selectivity of pheromone reception. It would be interesting to examine experimentally whether the pH around the olfactory pore and dendritic membrane are fine-tuned to optimize the uptake and release of physiologically relevant ligands. In addition to their function as odorant carriers and possibly odorant discriminators by acting as molecular filters for odorants crossing the sensillum lymph, PBPs might also play a role in the activation of odorant receptors (ORs) (Kaissling, 2001; Pophof, 2004). While it is largely accepted that PBPs solubilize, transport, and concentrate pheromones into sensillum lymph, which should enhance sensitivity to pheromone detection, it remains controversial whether PBPs may also help discriminate among pheromone components (see section Specificity of Pheromone Receptors).

### Sex pheromone receptors

Pheromones delivered at the dendritic membrane of ORNs are recognized by sex pheromone receptors. Insect OR genes were first identified in the fruit fly *Drosophila melanogaster* using a bioinformatics-based approach along with large-scale screening of olfactory tissue-specific genes encoding 7-transmembrane G-protein-coupled receptor (GPCR)-like proteins (Clyne et al., 1999; Gao and Chess, 1999; Vosshall et al., 1999). Those studies indicated that insect ORs form a unique gene family with no obvious homology to other proteins. The first identification of moth sex pheromone receptors from *B. mori* (Sakurai et al., 2004) revealed that sex pheromone receptors are members of the insect OR family. To date, moth sex pheromone receptors from 13 species have been reported (Sakurai et al., 2004; Nakagawa et al., 2005; Große-Wilde et al., 2007; Mitsuno et al., 2008; Forstner et al., 2009; Miura et al., 2009, 2010; Wang et al., 2010; Wanner et al., 2010; Zhang et al., 2010; Montagné et al., 2012; Xu et al., 2012a). These pheromone receptors are comprised in an isolated cluster within the OR family, suggesting that they evolved from a common ancestral gene. It is noted that not all ORs in this cluster would be sex pheromone receptors. For example, *EpOR1*, an OR from the light brown apple moth *Epiphyas postvittana*, is placed in this cluster. However, insect cultured cells expressing EpOR1 responded to plant-derived odorants such as methyl salicylate and a range of terpenes but not to sex pheromone components of female *E. postvittana* (Jordan et al., 2009).

In *B. mori*, 66 OR genes were found in the almost completely sequenced genome (The International Silkworm Genome Consortium, 2008; Tanaka et al., 2009). Of these, 5 ORs (*BmOR1, 3, 4, 5, 6*) are placed in the above-mentioned cluster and specifically or predominantly expressed in male adult antennae (Nakagawa et al., 2005). We previously demonstrated that *Xenopus* oocytes that co-express BmOR1 or BmOR3 and the *B*.*mori* OR co-receptor (BmOrco, originally named BmOR2), which can form heteromeric complexes with conventional ORs (see below), show specific responses to bombykol and bombykal, respectively (Nakagawa et al., 2005). In addition, these receptors are expressed mutually exclusively in ORNs in long s. trichodea (Krieger et al., 2005; Nakagawa et al., 2005). In contrast, neither bombykol nor bombykal activated the other 3 BmORs (BmOR4, 5, 6) (Nakagawa et al., 2005). These studies identified BmOR1 and BmOR3 as sex pheromone receptors in *B. mori*, and suggest that the specific detection of pheromone components by corresponding ORNs is accomplished by the strict molecular recognition of BmOR1 and BmOR3. However, there are contradictory reports regarding the specificity of these receptors, and therefore it remains unclear how the reception specificity of pheromone components is determined, as discussed below (see section Specificity of Pheromone Receptors).

### Sensory neuron membrane protein

A sensory neuron membrane protein (SNMP) (Vogt et al., 2009) that belongs to the CD-36 scavenger receptor family has been reported to be an essential molecular component of pheromone sensitivity in *D*. *melanogaster* (Benton et al., 2007; Jin et al., 2008). Interestingly, SNMP expression is required for the functional reconstruction of the sex pheromone receptor HvOR13 from the tobacco budworm *Heliothis virescens* in *D*. *melanogaster* pheromone-sensitive ORNs in sensillum trichodeum (Benton et al., 2007). This suggests that SNMP could be an essential molecular component of pheromone reception not only in *D*. *melanogaster* but also in moth species. There are at least two moth SNMPs (SNMP1 and SNMP2), of which SNMP1 is expressed in subsets of pheromone-sensitive ORNs whereas SNMP2 is expressed in accessory cells surrounding ORNs in *H*. *virescens* (Forstner et al., 2007). This differential expression pattern suggests that SNMP1 may play an important role in pheromone sensitivity in this moth species.

### Pheromone degrading enzyme

For the efficient orientation of male moths to conspecific females, the tracking of intermittent pheromone stimuli with high temporal resolution is required. For this, after activation of the pheromone receptors, pheromone molecules must be rapidly degraded into non-active substances to prevent from prolonged activation of the pheromone receptors. Antennae of *B. mori* possess alcohol oxidase (AOX) or alcohol dehydrogenase activity, which can oxidize bombykol into bombykal and bombykal into the inactive compound hexadecenoic acid (Rybczynski et al., 1990). Two AOX genes that are expressed predominantly in antennae and may possess this oxidizing activity have been cloned and named AOX1 and AOX2 (Pelletier et al., 2007). AOX2 may be responsible for pheromone degradation observed in antennae because it, and not AOX1, is expressed in the long s. trichodea of male moths. However, functional characterization of the AOX genes is required to better understand the mechanism of pheromone degradation and inactivation in *B. mori*. Regarding pheromone degradation by AOX, the isolation and functional characterization of an AOX2 homolog from navel orangeworm moth, *Amyelois transitella*, for pheromone degradation has recently been reported (Choo et al., 2013).

## Chemo-electrical signal transduction of odorants and sex pheromones

**Figure 2:**
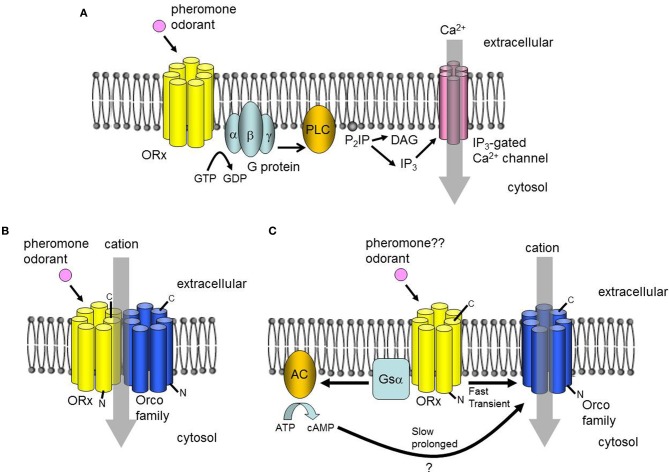
**Proposed signal transduction mechanisms. (A)** A classical model of insect olfactory transduction that involves a G-protein-mediated PLC-IP3 pathway. **(B)** Alternative model in which the odorant receptor (OR) forms a heteromeric odorant-gated non-selective cation channel with an Orco family protein. **(C)** Alternative model postulating two pathways, both of which depend on the cation channel function of Orco. An ionotropic pathway involves the direct activation of Orco by an OR with a bound ligand inducing a rapid but transient cation influx. A metabotropic pathway is coupled to the G protein, and induces slow but prolonged cation currents.

Upon binding to sex pheromone receptors, the chemical information of pheromones is converted into electrical signals in the ORNs. One of the most surprising findings in recent insect olfaction research may be the molecular mechanism by which ORNs convert chemical signals into electrical signals. Earlier studies reported a rapid and transient increase in inositol-triphosphate (IP_3_) in the antennae after pheromone stimulation, the expression of heterotrimeric G-proteins in the ORNs, and the activity of its effector enzyme in antennae homogenates (reviewed in Krieger and Breer, 1999; Jacquin-Joly and Merlin, 2004). These reports suggested that the odorant and pheromone receptors that transduce chemical signals into electrical signals were GPCRs, functioning via a heterotrimeric G-protein-mediated second messenger cascade (Figure 2A) (reviewed in Krieger and Breer, 1999; Jacquin-Joly and Merlin, 2004). However, recent physiological analyses of ORs including sex pheromone receptors have largely revised this view, by providing evidence that insect ORs form heteromeric complexes with Orco (originally named *Or83b* in *D*. *melanogaster*) (Neuhaus et al., 2005; Benton et al., 2006) and function as odorant-gated ion channels (Sato et al., 2008; Smart et al., 2008; Wicher et al., 2008). Orco is an insect OR but is unique in that it is highly conserved in insect species, whereas conventional ORs are highly divergent within and between species, and it is expressed in most ORNs, whereas conventional ORs are expressed only in specific subsets of ORNs (Vosshall et al., 2000; Krieger et al., 2003; Nakagawa et al., 2005). These observations suggest that Orco is not involved directly in odorant binding, but rather has a more general function in insect olfaction. Indeed, additional studies have revealed that Orco acts as a chaperone for conventional ORs to localize them to the dendritic membrane of ORNs, and also binds to conventional ORs to form a heteromeric OR complex (Larsson et al., 2004; Neuhaus et al., 2005; Benton et al., 2006).

Sato et al. (2008) co-expressed BmOR1 with BmOrco and other combinations of Orco family members and ORs in heterologous expression systems. Examination of the electrophysiological properties of Orco/OR complexes revealed that they act as pheromone/odorant-gated non-selective cation channels (Figure 2B). Interestingly, there was no evidence for elevated second messenger levels after stimulation with ligands of the expressed ORs, suggesting that there was no involvement of a G protein-mediated cascade in the activation of the Orco/OR complexes. This conclusion has been supported by pharmacological analyses of cultured cells co-expressing Orco and ORs from *D*. *melanogaster* (Smart et al., 2008). Meanwhile, Wicher et al. (2008) detected rapid transient and slow, prolonged ion currents in cultured cells co-expressing DmOrco and *D*. *melanogaster* ORs after stimulation with the ligands for the expressed ORs. The authors proposed that fast currents result from the direct activation of Orco by ORs, and that slow currents occur via the G protein-mediated activation of Orco (Figure 2C). Both models are consistent with the hypothesis that odorant signals are mediated by the odorant-induced channel activity of ORs/Orco complexes or Orco. Nevertheless, further studies are necessary to build a consensus of the roles of G-protein-mediated second messenger cascades in the reception of odorants and pheromones. The phosphorylation of Orco by protein kinase C activated by second messengers may enhance the response to odorants (Wicher et al., 2011; Getahun et al., 2013), suggesting that the second messenger system modulates responses to odorants through the phosphorylation of Orco. However, these two reports considered general ORs from *Drosophila* only. It will be interesting to determine whether this type of modulation occurs in sex pheromone receptors of *B. mori* and other moth species.

In *B. mori*, bombykol induces a slow but long-lasting elevation of cyclic guanosine monophosphate (cGMP) in the antennae of males but not females (Ziegelberger et al., 1990). The electrophysiological response to bombykol may be attenuated by perfusion of membrane-permeable dibutyryl cGMP into the lymph of long s. trichodeum, suggesting that cGMP contributes to the adjustment of sensitivity of bombykol-sensitive ORNs, and may play a role in adaptation to bombykol (Redkozubov, 2000). The BmOR1/BmOrco channel may be modulated negatively by cyclic nucleotides containing cGMP (Nakagawa and Touhara, 2013). Surprisingly, that study showed that cGMP acts on BmOR1 extracellularly; the extracellular application of non-membrane permeable cGMP downregulated the response of BmOR1/BmOrco-expressing oocytes to bombykol. Interestingly, this modulation was specific to BmOR1 and was not observed with BmOR3, *Ostrinia scapulalis* sex pheromone receptor, or other ORs tested.

Insect ORs and Orco represent a novel ion channel family with no known related protein sequences. In addition, insect ORs have a reversed topology compared to conventional GPCRs, with their N-terminus on the cytoplasmic side and the C-terminus on the extracellular side of the membrane (Benton et al., 2006; Lundin et al., 2007; Jordan et al., 2009). Therefore, the modes of action of this channel family are intriguing, and presently unclear. An important question is what is the subunit composition and stoichiometry of this ion-channel, and particularly whether conventional ORs can form part of the ion channel or if conventional ORs can activate ion-channels composed of Orco alone. Using *D. melanogaster* conventional ORs and Orco, Orco/Orco and OR/OR homomeric interactions have been also reported, in addition to OR-Orco heteromeric interaction described above (Neuhaus et al., 2005; German et al., 2013), though stoichiometry of the ion-channel is still unclear. Comprehensive mutation analyses of BmOR1 and BmOrco that targeted positively charged residues conserved in moth sex pheromone receptors and in the Orco family have revealed that mutation of 13 of 83 conserved residues alters the channel properties. In addition, 3 of the 13 residues (2 in BmOR1 and 1 in BmOrco) alter ion selectivity. This suggests that both conventional OR and Orco contribute to ion permeability, and that the OR/Orco complex functions as an odorant-gated ion channel (Nakagawa et al., 2012). Additional residues that are necessary for OR function have been identified using site-directed mutagenesis. Replacing two of four proline residues in the predicted second extracellular loop of BmOR1 with alanine significantly reduces the responsiveness to bombykol expressed in *Xenopus* oocytes (Xu and Leal, 2013). To further understand the roles of these residues, characterization of the three-dimensional structures of ORs and the OR/Orco complex is important.

## Specificity of pheromone receptors

Although the body of information on the molecular components involved in sex pheromone reception is increasing, how these factors achieve the high selectivity of ORN responses remains a major question in olfactory research. Regarding the specificity of *B. mori sex* pheromone receptors BmOR1 and BmOR3, three different observations have been reported. Nakagawa et al. (2005) showed that *Xenopus* oocytes expressing BmOR1 or BmOR3 together with BmOrco responded specifically to bombykol or bombykal, respectively, with only trace level responses of BmOR1-BmOrco expressing oocytes to a high dose of bombykal (300-fold higher dose than threshold dose for bombykol), suggesting that discrimination of the sex pheromone components is achieved by the expression of specific sex pheromone receptors in *B. mori*. In contrast, Xu et al. (2012b) reported that *Xenopus* oocytes co-expressing BmOR1 and BmOrco responded to both bombykol and bombykal. Since sensitivity to bombykal was only one order-of magnitude lower than that to bombykol, Xu et al. concluded that “the responses of the “naked receptor” differ from the neuronal activity of the olfactory system of the silkworm” (Xu et al., 2012b, p. 2). Grosse-Wilde et al. (2006) revealed that HEK293 cells expressing BmOR1 with a heteromeric Gα15 subunit responded to both bombykol and bombykal when these compounds were dissolved in dimethyl sulfoxide (DMSO). In contrast, when BmPBP1 was used to solubilize these compounds, BmOR1- and Gα15-expressing HEK293 cells responded to bombykol specifically. Based on these results, Grosse-Wilde et al. (2006) proposed that BmPBP1 binds and solubilizes bombykol but not bombykal and that the interplay between a specific PBP and the receptor determines the specificity of the pheromone reception system. This hypothesis implies that PBPs that bind selectively to bombykal are present in sensillum lymph of long s. trichodeum. To assess this, two OBPs that belong to the PBP subfamily were isolated but were expressed in different cells from the BmPBP1-expressing ones, demonstrating that these PBPs function in non-trichodeum sensilla (Forstner et al., 2007). Zhou et al. (2009) reported that BmGOBP2, a member of the *B. mori* OBP family, has different binding affinities for bombykol and bombykal; BmGOBP2 bound bombykol more than bombykal. However, BmGOBP2 is not expressed in male long s. trichodeum (Maida et al., 2005) and thus is unlikely to mediate pheromone signals in male antennae. The discrepancies between these studies may be due to the different expression systems and/or experimental conditions. Therefore, *in vivo* analyses of the genes involved in sex pheromone detection using knockout or transgenic techniques are crucial to unequivocally determine whether receptor specificity alone is sufficient to explain ORN specificity, or whether additional components such as BmPBP1 are also required.

## Antennal lobe

**Figure 3:**
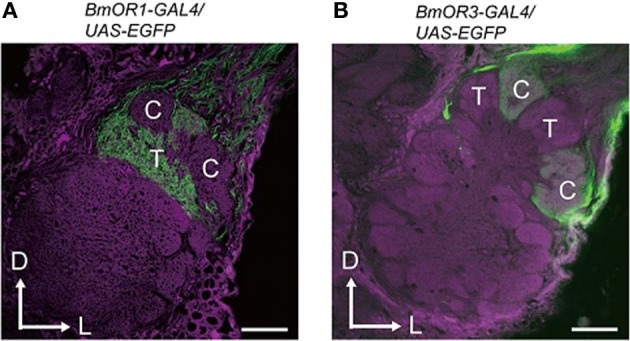
**Projection patterns of bombykol- or bombykal-sensitive ORNs to the AL.** The axon terminals of bombykol **(A)** and bombykal **(B)** receptor neurons were labeled by EGFP using the BmOR1 or BmOR3 promoter-GAL4 lines and a UAS-EGFP line. EGFP was visualized using anti-GFP immunostaining (green). Background staining was carried out using Alexa Fluor 555 (magenta). Representative confocal sections are shown. C, cumulus; T, toroid; D, dorsal; L, lateral. Scale bars: 50 μm. (modified from Sakurai et al., 2011).

**Figure 4:**
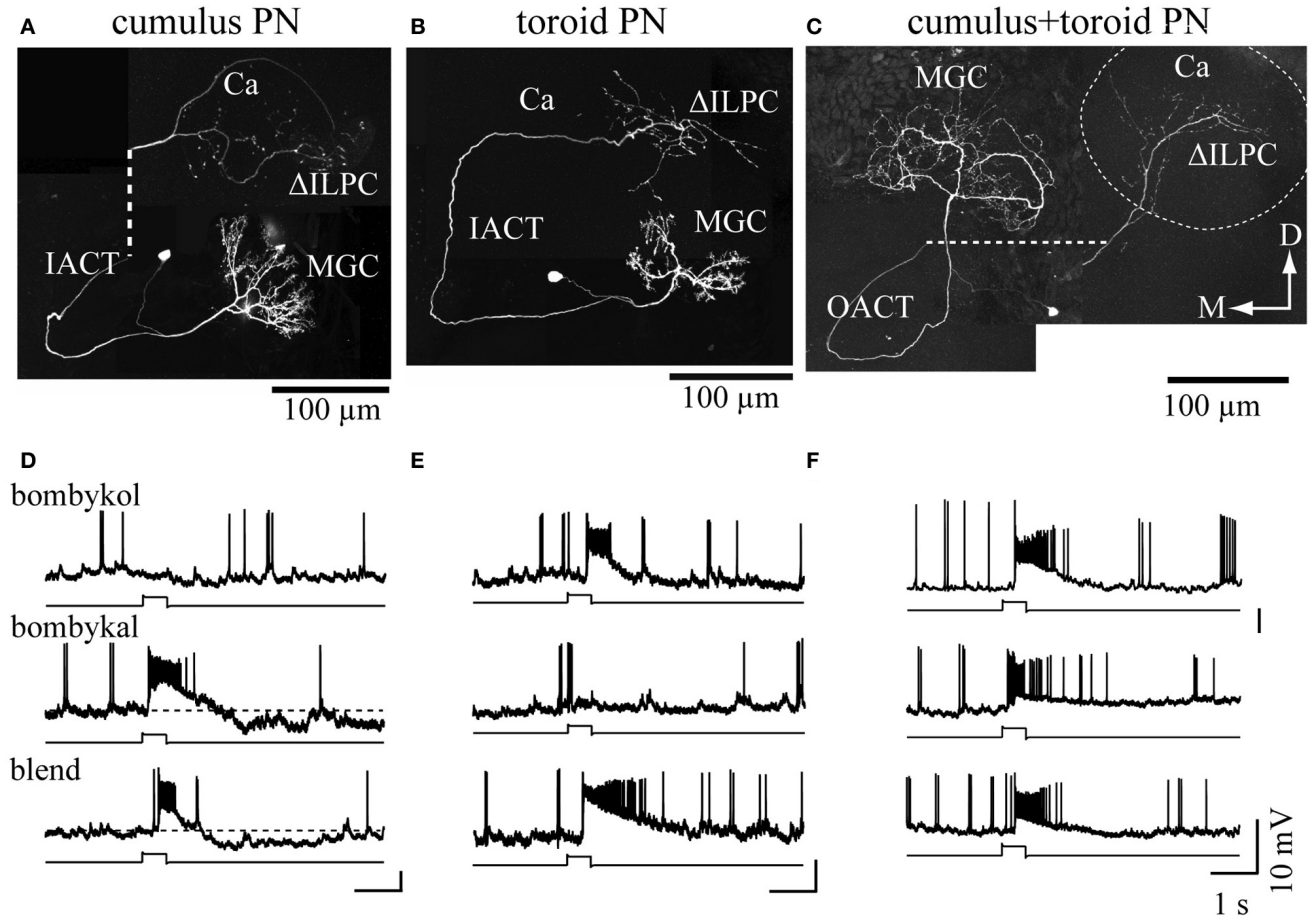
**Combinatorial representation of pheromones in the macroglomerular complex in the antennal lobe. (A–C)** The morphologies of projection neurons innervating the cumulus **(A)**, toroid **(B)**, and both subdivisions **(C)**. The dashed line in **(A)** and **(C)** links processes that were separated to reveal the structures of branches that overlapped *in situ*. The dashed circle in **(C)** indicates the area of calyx of the mushroom body. **(D–F)**. The physiology of the projection neurons innervating the macroglomerular complex. The response properties shown in **(D–F)** correspond to **(A–C)**, respectively. Changes in the membrane potential in response to 200 ms odor presentation are shown. Bombykol, bombykal, and a combination of the two components were examined. Neurons innervating the toroid or cumulus showed selectivity in their odor-response properties, whereas multiglomerular neurons innervating both the toroid and cumulus responded to all stimuli. Ca, calyx of the mushroom body; D, dorsal; IACT, inner antennocerebral tract; M, medial; MGC, macroglomerular complex; OACT, outer antennocerebral tract; ΔILPC, delta area of the inferior lateral protocerebrum. (modified from Kanzaki et al., 2003).

Pheromone signals that are transduced into electrical signals by ORNs are transmitted to the primary olfactory center, the AL, through the axons of ORNs. The AL is divided into two regions: the dorsally located macroglomerular complex (MGC), and the ventrally located ordinary glomeruli (OG). The MGC receives pheromone inputs from pheromone-sensitive ORNs on the antennae, and have several compartments named the toroid, cumulus, and horseshoe (Koontz and Schneider, 1987). The AL has two types of interneurons: local interneurons (LNs) that connect to the glomeruli, and projection neurons (PNs) that receive input from the glomeruli and send processed information to the higher-order olfactory centers. In *B*. *mori*, the genetic labeling of ORNs that express BmOR1 or BmOR3 has revealed that bombykol and bombykal receptor neurons project convergently to the toroid and cumulus, respectively (Figure 3) (Sakurai et al., 2011). PNs innervating the toroid and cumulus then respond selectively to bombykol and bombykal (Kanzaki et al., 2003). Therefore, each compartment processes information for different components (Kanzaki et al., 2003). High response selectivity of PNs have been observed in other species (Hansson et al., 1991; Heinbockel et al., 1999, 2004; Schlief and Wilson, 2007). When a combination of bombykol and bombykal is present, PNs of both the toroid and cumulus exhibit an excitatory response, suggesting combinatorial representation of the pheromone components (Figure 4).

The OG is a group of 60 relatively small glomeruli. Each individual glomerulus receives inputs from the sensilla basiconica or the sensilla coeloconica. Due to the simplicity of the insect olfactory system, individual glomeruli are identifiable using an anatomical procedure with a digital atlas of the AL (Rospars, 1983; Galizia et al., 1999; Kazawa et al., 2009), because they all exhibit characteristic volumes, shapes, and/or positions. In addition, the position and shape of non-neuronal structures such as neuronal bundles and cell clusters can easily be discriminated. Using these prominent anatomical structures as landmarks allows each individual glomerulus to be identified (Kazawa et al., 2009).

LNs usually exhibit an axon-less shape. Consistent with other species (Matsumoto and Hildebrand, 1981; Chou et al., 2010), *B*. *mori* shows significant variability in the morphology of LNs. We have performed comprehensive samplings of LNs in the lateral cell cluster of the AL, and have analyzed them systematically (Seki and Kanzaki, 2008). Based on the analysis of 153 neurons, we classified LNs with distinct morphological characteristics such as the number of innervating glomeruli, dendritic density. The most prominent are the multiglomerular type which innervate most glomeruli, including the MGC (MGC-allGs-sparse). The another multiglomerular type, MGC-allGs-dense LNs also innervate most glomeruli, and exhibit a dense arborization in the OG but a sparse arborization in the MGC. Thirty-five out of total 153 were oligoglomerular LNs. This group is further separated by the presence or absence of innervation into MGC: oligoglomerular LNs only innervating OG (oligoGs) and those with innervating both MGC and OG (MGC-oligoGs). MGC oligoGs LNs exhibit arborization in the MGC and 10–60% of the OG, and show a high level of morphological variation. Most LNs, except for some MGC-oligoGs LNs, show a GABA-like immunoreactivity. Diverse morphologies of LNs have also been reported in *Manduca sexta* (Reisenman et al., 2011).

PNs usually have densely aggregated dendritic structures and long axons that protrude to the higher order centers. In a comprehensive sampling of the 246 PNs that run through five different neuronal tracts that are homologous to *M*. *sexta* (Homberg et al., 1988; Kanzaki et al., 1989), we revealed that 76% of the total number of PNs were uniglomerular. Furthermore, this percentage increased to 83% for PNs innervating the OG (Namiki and Kanzaki, 2011). The relative volume of PNs is higher in OG than in the MGC. This is partially due to the difference in the number of PNs between the MGC and OG. We estimate that the toroid in the MGC contains ~40 PNs, and that each individual glomerulus in the OG contains ~3.5 PNs. The higher numbers of ORNs and PNs thus augment MGC volume.

Most PNs receive input from only a single glomerulus, which supports the hypothesis that glomerulus output can be monitored by following the activity of individual PNs. Therefore, we examined the odor response properties of individual PNs and reconstructed AL activity using a digital atlas of responses to plant odors (Namiki and Kanzaki, 2008). We translated the firing rates of individual PNs into color codes and then plotted them to the atlas. In the AL output, plant odors evoked distinct spatial patterns, which changed with time. Plant odor information was encoded by spatially and temporally organized activity patterns among OG PNs, whereas pheromones were represented in a combinatorial fashion by MGC compartments.

## Amplification of the ORN input in the AL

In several moth species, the sensitivity of a pheromone-responsive PN is much higher than an ORN tuned to the same pheromone (Hartlieb et al., 1997; Jarriault et al., 2010). Therefore, pheromone signals are likely to be amplified during processing in the AL. This amplification may be explained, in part, by a high convergence ratio between ORNs and PNs (Jarriault et al., 2009). For example, each antenna of male *B*. *mori* possesses ~17,000 bombykol-sensitive ORNs that project to ~40 PNs. Therefore, pheromone signals that evoke very weak activities in many ORNs may be spatially integrated by PNs to elicit strong neural activities. In addition to this amplification, by using an optogenetic approach with transgenic silkmoths expressing a blue light-sensitive ion channel (channelrhodopsin-2 [ChR2] from the green algae *Chlamydomonas reinhardtii*) only in BmOR1-expressing ORNs, we recently found that weak ORN inputs that are sub-threshold in terms of behavior, but not strong ORN inputs, can be amplified to supra-threshold levels by temporal integration in PNs (Tabuchi et al., 2013). Pharmacological analyses revealed that the integration is likely to be an intrinsic property of PNs, and that GABAergic mechanisms inhibit the integration of strong ORN inputs. Interestingly, the time window for the temporal integration (<80 ms) is close to the average duration of a single odor filament in an odor plume under natural conditions (Murlis et al., 2000), suggesting that male *B. mori* use this mechanism to detect very weak environmental pheromone signals.

## Lateral protocerebrum

**Figure 5:**
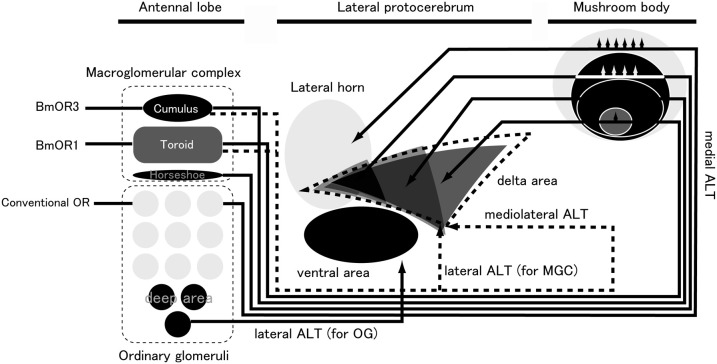
**Pheromone-processing pathway in the sensory system of *B*. *mori*.** ALT, antennal lobe tract; MGC, macroglomerular complex; OG, ordinary glomeruli. (modified from Namiki et al., 2013).

PNs receive olfactory inputs in the AL and send processed information to the higher-order centers of the lateral protocerebrum (LPC) and the calyx of the mushroom body (MB) (Figure 5). The LPC is thought to be the center for instinctive behavior because the wiring in the LPC is independent of sensory input (Tanaka et al., 2004). In *Drosophila*, chemical ablation of the MB by using food containing hydroxyurea for newly hatched larvae does not affect mating behavior (Kido and Ito, 2002). The AL PNs project to the stereotypic locus in the LPC, forming a map of olfactory inputs from the AL (Marin et al., 2002; Wong et al., 2002; Nishino et al., 2003; Tanaka et al., 2004). In *B. mori*, PNs for processing sex pheromone project distinct area than the other PNs. Seki et al. (2005) stained the PNs that process bombykol information via immunocytochemistry with anti-cGMP antibodies. By simultaneously staining individual PNs and antibodies, they created an olfactory map of the *B. mori* LPC. Toroid PNs project selectively to the delta area of the inferior lateral protocerebrum (ΔILPC). As a minor pheromone component, bombykal is processed in the cumulus of the AL, and then is sent to the lateral part of the ΔILPC, which overlaps partially with the area of axonal projection of toroid PNs. OG PNs send axonal projections to the lateral portion of the LPC, and there seems to be no overlap between OG PNs and MGC PNs. Additional OG PNs that innervate the posterior region send axonal projections to the ventral part of the LPC, which is different from the projection areas of other OG PNs. In the fruit fly, the structures of deep glomeruli are also distorted compared to other glomeruli. PNs from these glomeruli running through the medial AL tract (corresponding to inner antennocerebral tract in *B. mori*; Ito et al., 2014) innervate a similar area to other PNs in the LH, and PNs running through the lateral AL tract innervate a ventral area in the protocerebrum (Grosjean et al., 2011). In the cockroach AL, PNs that innervate deep glomeruli have been characterized, and they respond to humidity and temperature (Nishino et al., 2003). These PNs run through the lateral ALT (lALT, corresponding to the outer antennocerebral tract in *B. mori*) and send axonal projections to the protocerebrum, which is located ventral to the LH. The structures of these glomeruli are also distorted. Therefore, the axonal projections of AL PNs seem to be divided into three areas: the ΔILPC, the lateral part of the LPC, and the ventral part of the LPC (Namiki and Kanzaki, 2011).

Previous studies have revealed several functional roles of the LPC for sensory processing, including switching courtship behavior (Broughton et al., 2004), repulsive behavior (Wang et al., 2003), and multimodal integration (Nishino et al., 2003). Anatomical studies on *Drosophila* in which antennae were cut have suggested that spatial mapping in the LPC is innately programmed, indicating that this circuit is involved in innate behavior (Heimbeck et al., 2001; Tanaka et al., 2004). For example, avoidance behavior may be innately programmed and it requires fast information processing. This view is consistent with a previous anatomical study that focused on the innervation of descending interneurons in the cockroach brain (Okada et al., 2003). The LH is innervated directly by descending interneurons in the cockroach, which may result in faster processing than the pathway through the MB (Okada et al., 2003).

The functional role of ΔILPC in pheromonal processing is unclear. It is possible that the function of this circuit is innately programmed and less plastic, such as the integration of bilateral information or blended-ratio discrimination. A recent study identified mixed-ratio-sensitive neurons innervating the LPC (Lei et al., 2013), suggesting a functional role in cognate pheromone recognition. Blended-ratio discrimination is important for mating success and evolution. The systematic examination of mixtures of pheromone components with different ratios may provide a better understanding of this issue.

## Mushroom body

The MB is thought to be the center of learning and memory (Heisenberg, 2003). However, there is no clear experimental system for investigating learning and memory in *B. mori*. Several behavioral experiments have suggested an important role of the MB in pheromone processing. The MB of *B. mori* is located in the dorsal part of the protocerebrum, and is divided into three structures: the calyx, pedunculus, and lobes. Consistent with other moth species, a second tract called the Y tract originates in the dorsoanterior region of the calyx, and projects to the branching point of the vertical and medial lobes.

We analyzed the anatomical organization of the MB in *B. mori*, and performed comprehensive sampling of the Kenyon cells (KCs), the intrinsic cells in the MB (Fukushima and Kanzaki, 2009). Based on staining of 109 of ~2000 KCs, we classified KCs into four types according to the axonal projections in the lobes: α/β, α^'^/β^'^, γ, and Y KCs. The α/β KCs have dendritic arborization in the medial or lateral half of the calyx, with numerous spiny specialized structures; they do not invade the posterior portion. The neurons send forked axonal processes with complicated side branches bearing small varicosities to the α/β lobe. α^'^/β^'^ KCs have dendritic arborization in a large area of the calyx via numerous spiny specialized structures similar to those of α/β neurons, and send axonal processes to the α^'^/β^'^ lobe. The axonal processes in the lobe have complex collaterals bearing small varicosities. The γ KCs have an elongated narrow dendritic field in the calyx with claw-like structures, and send axonal projections to the γ-lobe; they have sparse and short collaterals with curling branches at the tip. The α/β, α^'^/β^'^, and γ neurons also have side branches in the peduncle. Y KCs also have a small dendritic field predominantly in the anterior part of the calyx, and send axonal projections to the ventral, dorsal, and medial lobules of the Y division running through the Y-tract.

The inputs from the AL form a map in the calyx (Namiki et al., 2013) (Figure 5). Different projection areas for pheromonal and non-pheromonal PNs have been reported in the calyx of moths. The inputs have a concentric spatial pattern: information in the toroid, cumulus/horseshoe, and OG is ordered from the center to the edge, suggesting that pheromones and plant odors are processed differently in the calyx than in the LPC. PNs from the cumulus, horseshoe, and OG also overlap significantly. The existence of a modular structure in the calyx is supported by other types of LPC neurons that also innervate the calyx in a class-specific manner.

In our study, each class of PN showed functional connectivity to KC classes with different patterns in *B*. *mori* (Namiki et al., 2013). The α/β and α^'^/β^'^ KCs exhibited large branches in the calyx. Because they showed a high level of potential connectivity with all PN classes, they could integrate information from sex pheromones and plant odors. This dense connectivity is consistent with reports from locusts. An electrophysiological study in the locust demonstrated that PNs connect densely to KCs, and that a single KC has synaptic connections with about half of the number of PNs (Jortner et al., 2007). γ and Y KCs have dendrites in a small area of the calyx, which suggests that they may be able to extract specific types of odor information. We hypothesize that α/β and α^'^/β^'^ KCs receive a broader input (including sex pheromones) compared to γ and Y KCs. Based on their potential connectivities, these KCs may integrate pheromonal and non-pheromonal information.

No KCs have a dendritic field that is restricted to the input area of toroid PNs, suggesting that pure bombykol information is not preserved at the level of third-order neurons in the MB. Therefore, the calyx may not be involved in the labeled-line for pheromone coding, although it is possible that the pheromone response in KCs affects some aspects of mating behavior. The odor response of KCs is sparse in *B. mori*, consistent with other insects, but at least α^'^/β^'^ KCs are responsive to sex pheromones (unpublished observation, Shigehiro Namiki and Ryohei Kanzaki), and bombykol induces neural activity in cells around the calyx that are thought to be KCs (Fujita et al., 2013).

## Processing multicomponent pheromones

Many moth species use a set of multiple compounds as their sex pheromone, and some species respond only to specific ratios of such multicomponent pheromones. There are several possibilities to explain the neural mechanisms for mixture recognition. First, the postsynaptic neurons might integrate component information from individual PNs. For example, synchronization between PNs could encode ratio information. Salient olfactory stimuli on a single glomerulus in the MGC can affect the activity of an adjacent one via LNs (Lei et al., 2002). The stimuli do not inhibit the number of spikes, but rather facilitate the synchronization between PNs innervating adjacent glomeruli. They also do not affect the firing rate, but rather the degree of synchronization reports the ratio information of the pheromone components. In *Manduca*, the activities of PNs are more synchronized in cognate mixtures of sex pheromones (2:1) (Martin et al., 2013). Synchronized activity can also be detected in downstream neurons in the protocerebrum (Lei et al., 2013).

Another possibility for the recognition of pheromones are the multi-glomerular PNs that innervate more than two glomeruli (Homberg et al., 1988; Kanzaki et al., 2003), running through different neuronal pathways to uni-glomerular PNs. Multi-glomerular PNs that show synergistic responses to pheromone mixtures have been reported in several species (Christensen and Hildebrand, 1987; Wu et al., 1996; Anton et al., 1997).

In *B. mori*, bombykal suppresses the behavioral response to bombykol (Kaissling et al., 1978; Daimon et al., 2012a), but the function of the minor pheromone component remains unknown. Bombykal is thought to be ancestral pheromone of *Sphingidae*, and many species use it as their sex pheromone (El-Sayed, 2011). Some closely related *Bombycidae* species also use bombykal as a pheromone component (Daimon et al., 2012b), and a comparison between *B. mori* and these species could be helpful to understand the processing of multicomponent pheromones.

## Lateral accessory lobe

The lateral accessory lobe (LAL) is a neuropil located in the ventral portion of the protocerebrum that is thought to be important for moth orientation behavior in response to the pheromone source. Descending neurons (DNs), which show alternating firing responses to sex pheromones, have a dendritic branch in the LAL (Kanzaki et al., 1994; Mishima and Kanzaki, 1999; Wada and Kanzaki, 2005). The alternating activity pattern is referred to as “flip-flop” between interneurons, after the basic concept of “memory” in electronic circuit operation (Olberg, 1983; Kanzaki et al., 1994). There are two types of interneurons in the LAL: unilateral interneurons that innervate LAL on one hemisphere, and bilateral interneurons that innervate the LAL on both hemispheres. According to the immunoreactivity of LAL commissure, there are 4 serotonergic, ~40 GABAergic, and 20 other bilateral neurons. LALs are also connected to other areas in the protocerebrum including the central complex (in locusts, Homberg, 1994; Li et al., 2009; flies, Phillips-Portillo, 2012), anterior optic tubercle (in locusts, Homberg, 1994; Homberg et al., 2003), and the superior median protocerebrum (in locusts, Homberg, 1994; Homberg et al., 2003; Li et al., 2009; flies, Phillips-Portillo and Strausfeld, 2012; Lin et al., 2013). The LAL can be further divided into two regions: the inner and outer parts. LAL neurons often innervate an adjacent unstructured neuropil called the ventral protocerebrum (VPC). The VPC is divided into three sections: the inner VPC, the anterior part of the inner VPC, and the outer VPC (Iwano et al., 2010). When we assessed the responsiveness of LAL neurons to pheromone stimulation at the left and right antennae of *B. mori*, the neurons showed similar response patterns in terms of intensity, latency, and duration (Namiki and Kanzaki, 2012).

DNs have been characterized using intracellular recording methods, which have revealed that individual DNs have distinct physiological and morphological features (Mishima and Kanzaki, 1999; Wada and Kanzaki, 2005). Group I DNs have their soma on the anterior surface of the brain, and are located ventral to the medial cell cluster in the AL. Among these DNs, type A and B neurons innervate the LAL. Group II DNs have their soma on the anterior surface of the brain, and are located dorsal to the AL. Among these DNs, type A and D neurons innervate the LAL. In addition, groups IA, IIA, and IID DNs show flip-flop activity, which resents as persistent firing activity when they receive transient pheromone input. The persistent firing becomes silent when the neuron receives additional pheromone input. Because all flip-flop neurons in the supraesophageal ganglion that have been identified so far innervate the LAL, the LAL is thought to be the premotor center for pheromone-mediated walking behavior. Group III DNs have their soma in the posterior part of the brain, and although some group III DNs respond to pheromones with transient excitation, long-lasting activity has not yet been observed, supporting the hypothesis that LAL output is the source of command information for orientation behavior.

The organization of DNs has been described in several insect species (Strausfeld et al., 1984; Staudacher, 1998; Okada et al., 2003; Cardona et al., 2009). Most DNs originate in the posterior and ventral part of the brain, and have their cell bodies on the posterior brain surface. In *Drosophila*, anterior-located group I and group II DNs, and posterior-located group III DNs might correspond to ventral and dorsal DNs, respectively. Dorsal DNs project to the dorsal neuropils in thoracico-abdominal ganglia, whereas all known ventral DNs project to ventral neuropils (Milde and Strausfeld, 1990; Strausfeld and Gronenberg, 1990). The central complex is thought to play an important role in locomotor behavior (Strauss, 2002; Strausfeld and Hirth, 2013), transmitting premotor information to thoracic motor centers. Anatomical studies have suggested that the LAL is the major output site for the central complex (Kim et al., 2013; Lin et al., 2013). Although the properties of DNs innervating the LAL are less well defined in other insect species, we speculate that the function of LAL DNs in the control of locomotion is not specific to *B. mori* but rather is common to insect brains across different species. DNs innervating the LAL with similar morphology as pheromone-processing DNs have also been reported in some insect species; for example, group-I-like cells in crickets (Zorović and Hedwig, 2011, 2013) and group-II-like cells in flies (Yu et al., 2010). DNs similar to group I have been reported to respond to visual and auditory stimuli (Olberg, 1986; Brodfuehrer and Hoy, 1990; Gonzalez-Bellido et al., 2013). In ants, LAL DNs, whose morphologies are similar to *B. mori* group-I DNs, show a longer excitatory response to pheromones than to plant odors (Yamagata et al., 2007). In crickets, the activities of LAL DNs are correlated with forward and/or rotational velocity (Staudacher, 2001), and the activation of a single DN can trigger walking (Zorović and Hedwig, 2013).

## Hard wiring between ORN and behavioral responses

**Figure 6:**
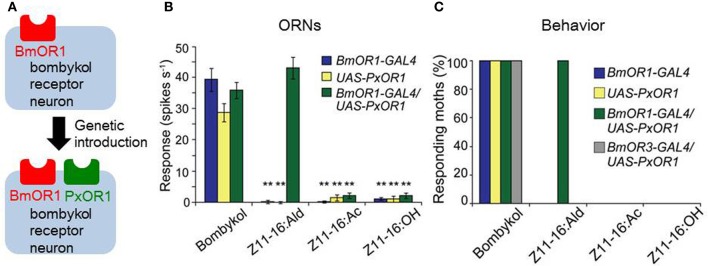
**Labeled-line coding of bombykol information. (A)** A GAL4 line with putative BmOR1 promoter sequences (BmOR1-GAL4) and an effector line expressing PxOR1 under the control of the UAS sequence were generated, and crossed to induce the expression of PxOR1 in bombykol-sensitive ORNs. **(B)** Single sensillum responses of bombykol-sensitive ORNs. Bombykol-sensitive ORNs of BmOR1-GAL4/UAS-PxOR1 that express PxOR1 responded to *Z*-11-hexadecenal (Z11-16:Ald) as well as bombykol. *Z*-11-hexadecenyl acetate (Z11-16:Ac) and *Z*-11-hexadecenol (Z11-16:OH) are pheromone components of the diamondback moth, but are not ligands for PxOR1. ^**^p < 0.01 compared with responses of the corresponding line stimulated with bombykol; Scheffe's F test. **(C)** Percentages of male moths that exhibit pheromone-source searching behavior. Artificial activation of bombykol-sensitive ORNs by Z11-16:Ald triggered the behavior, demonstrating that the activation of BmOR1-expressing bombykol-sensitive ORNs is sufficient for the initiation of pheromone-source searching behavior. Expression of PxOR1 in bombykal-sensitive ORNs by a BmOR3 promoter-GAL4 line (BmOR3-GAL4/UAS-PxOR1) did not confer behavioral responsiveness to Z11-16:Ald. (modified from Sakurai et al., 2011).

Because of the strong correlations among pheromone chemicals, their ORNs, and behavioral responses, sex pheromone information has long been thought to be coded by a particular neuronal pathway (labeled line), particularly in species such as *B. mori* that rely on a single pheromone component to elicit behavior. Characterization of the receptor genes for sex pheromones together with the advancement of genetic manipulation techniques in *B. mori* (Tamura et al., 2000; Imamura et al., 2003) have enabled the experimental assessment of this long-held hypothesis. Our group generated a transgenic silkmoth line that ectopically expresses the sex pheromone receptor PxOR1 from the diamondback moth *Plutella xyllostella* specifically in BmOR1-expressing ORNs (Figure 6A) (Sakurai et al., 2011). Ectopic expression of PxOR1 allowed bombykol-sensitive ORNs to respond to *Z*-11-hexadecenal, a ligand specific for PxOR1 (Figure 6B). The artificial activation of BmOR1-expressing neurons by *Z*-11-hexadecenal triggered full sexual behavioral responses, including pheromone-source searching and copulation attempts with female moths (Figure 6C). Therefore, this provided direct evidence that the activation of BmOR1-expressing bombykol sensitive ORNs is sufficient to trigger sexual behavior. Using a transgenic silkmoth line that expressed a ChR2 only in BmOR1-expressing neurons, we demonstrated that the activation of BmOR1-expressing neurons by ChR2 with blue light is sufficient to elicit pheromone-source searching behavior, further supporting our findings (Tabuchi et al., 2013)

Analyses of silkmoths with a null mutation in *Bmacj6*, a homolog of *D*. *melanogaster abnormal chemosensory jump6* (*acj6*) that encodes a class IV POU domain transcription factor, have provided insight into the genetic mechanisms that may modify pheromone specificity at the behavioral level in moths (Fujii et al., 2011). In *D*. *melanogaster*, the *acj6* mutant displays abnormal olfactory behavior (Ayer and Carlson, 1991), and genetic and electrophysiological analyses have suggested that *acj6* determines the OR gene choice and axon targeting of ORNs (Clyne et al., 1999; Komiyama et al., 2004). Fujii et al. (2011) found that the expression of BmOR1 transcripts in male *Bmacj6* mutant antennae was ~1000-fold lower than in wild-type males. Consistent with lowered BmOR1 expression, electroantennogram and the behavioral responses to bombykol were significantly reduced in this mutant moth strain. Surprisingly, *Bmacj6* mutation not only reduced the behavioral responses to bombykol but also conferred behavioral responsiveness to bombykal. Because the response selectivity of PNs, second-order olfactory neurons, and innervating toroid changed from bombykol to bombykal, the authors hypothesized that the modified behavioral preference was caused by either the mistargeting of BmOR3-expressing ORNs into toroid that normally received information from bombykol receptor neurons, or the ectopic expression of BmOR3 in BmOR1-expressing ORNs. Although the understanding of the exact mechanisms of *Bmacj6* mutation requires further clarification, it should be noted that the modification of behavioral preferences did not require processing in the brain, but was determined at the peripheral level. From a viewpoint of the evolution of pheromone communication, it is of great interest to determine whether the *acj6* mutation was causally related to the modification of the pheromone preference of male moths during the evolution of moth species.

## Modification of programmed behavior

Although olfaction alone is sufficient to localize the odor source, several additional factors can affect the locomotion pattern of odor-searching behavior. For example, pheromone responsiveness and brain activity are modified by pre-exposure to ultrasonic songs produced by predators (Anton et al., 2011). Next, we introduce two examples of modified mating behavior in *B. mori*: the effects of odors other than pheromones, and the effects of neuromodulators on pheromone processing.

### Effects of non-pheromonal odors

Plant odors and insect semiochemicals interact under natural field conditions. Plant odors affect several important aspects of insect behavior such as pheromone production, release, and orientation behavior (Reddy and Guerrero, 2010, 2004). Although orientation behavior is innate, there are many influential internal and external factors including age, mating experience, and the concentration of juvenile hormone and serotonin in the brain (Anton et al., 2007; Kloppenburg and Mercer, 2008). The sensitivity of insect orientation behavior to sex pheromones is usually modified by host plant odor, and as such many species exhibit modified sensitivity in response to plant odors, as found in moths (Dickens et al., 1990; Reddy and Guerrero, 2010), beetles (Phillips et al., 1984; Byers et al., 1990), and flies (Landolt et al., 1992). At the peripheral level, modifications of pheromone responses have been characterized using electroantennograms (Plettner and Gries, 2010). In real environments, pheromones do not exist alone, but are present with other odors, including plant odors, that affect the sensitivity to pheromones. Such sensitivity to plant odor is exploited in pheromone trap systems, as the trapping score increases when plant odors are added (Light et al., 1993). In the corn earworm moth, *Helicoverpa zea*, the response of ORNs to the major pheromone component, (*E,E*)-8,10-dodecadien-1-ol, is elevated when the host plant odor, linalool is delivered as a mixture along with pheromone (Ochieng et al., 2002). Recent studies have revealed that such modulations of ORN activity may result from interactions between sex pheromone receptors and plant odorants. HvOR13 is a receptor for the major sex pheromone *Z*-11-hexadecenal (Z11-16:Ald) in *Heliothis virescens* (Große-Wilde et al., 2007). Stimulation with Z11-16:Ald in a mixture with some host plant odorants inhibits the calcium response in the cumulus of the MGC, which processes Z11-16:Ald information. Consistent with this, the Ca^2+^-response of HvOR13-expressing HEK293 cells to Z11-16:Ald is significantly reduced in the presence of plant odorants that inhibit calcium responses in the AL, whereas these plant odorants do not affect the binding of Z11-16:Ald to the corresponding PBP. Therefore, inhibition of pheromone receptors by plant odorants may modulate the physiological responses of pheromone receptor neurons (Pregitzer et al., 2012). The mechanisms at the receptor level might also underlie the increment of spiking activity observed in *H. zea* (Ochieng et al., 2002). Systematic study with a variety of plant odors is required for understanding the mechanisms for plant-odor effect on pheromone response.

**Figure 7:**
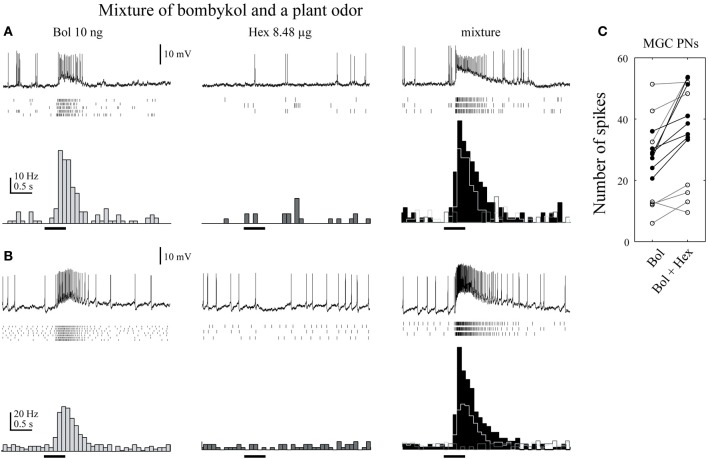
**A plant odor enhances the activity of toroid projection neurons in response to bombykol. (A)** Physiology of uniglomerular projection neurons innervating the toroid. Raster plots of individual trials (top) and the number of spikes (bottom) are shown. The neurons showed a transient excitatory response to bombykol (Bol), no response to *cis*-3-hexen-1-ol (Hex), and a transient excitatory response to the combination. The firing rate was higher in response to the combination than to bombykol alone. **(B)** Physiology of multiglomerular projection neurons innervating both the toroid and cumulus. The firing rate was higher in response to the mixture than to bombykol alone. **(C)** Population data on the effect of adding *cis*-3-hexen-1-ol. Data from 17 neurons are shown. (modified from Namiki et al., 2008).

Similar modulatory effects have been observed in *B. mori*. A mixture of bombykol and *cis*-3-hexen-1-ol increases the sensitivity of males to bombykol, whereas a mixture of bombykol and citral, another component of mulberry leaves, does not. When we recorded the membrane potentials of toroid PNs, there were significantly more spikes in response to mixtures than pheromone alone, providing evidence of a correlation between central brain activity and behavior (Namiki et al., 2008) (Figure 7). Neuronal correlates of behavioral modification by plant odors, including the suppression on pheromone processing in *Agrotis ipsilon* have been reported (Chaffiol et al., 2012; Deisig et al., 2012). In addition, the response patterns of OG PNs to plant odors are not different from the patterns seen in response to the mixture of plant odors and pheromone, suggesting that the discrimination system of moth plant odors is not affected by sex pheromones (Namiki et al., 2008). Therefore, moths seem to be able to process pheromones and plant odor information at the same time.

### Effects of serotonin

Several developmental phenomena are regulated by circadian rhythm in *B. mori*, including egg hatching and adult eclosion. The calling time of females also changes with circadian rhythm as well as reproductive behavior. Female moths have long calling times during the photophase, and they lay their eggs during the early scotophase (Yamaoka et al., 1976; Sasaki et al., 1984). Male moths, which are completely isolated from females in the absence of sex pheromones, sometimes show spontaneous wing fluttering during the photophase (Sasaki et al., 1984). Serotonin levels in the brain also change during the day, and the dynamics are similar to those of pheromone sensitivity in male moths (Gatellier et al., 2004); in that study, injecting 10^−4^ M serotonin into the brain increased the sensitivity of male moths to pheromones.

**Figure 8:**
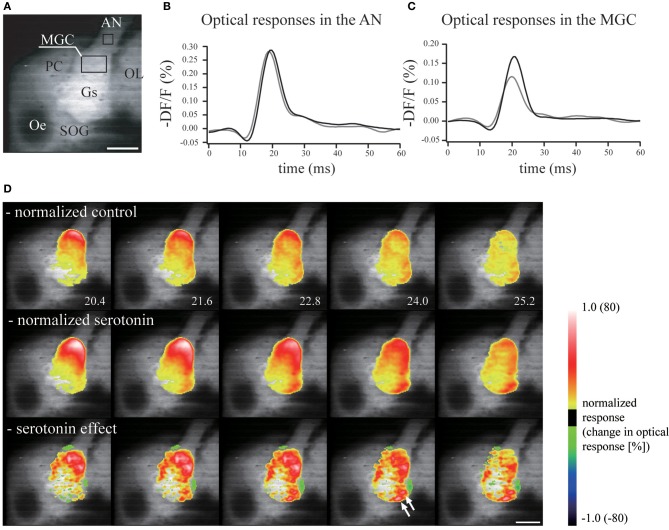
**Serotonin enhances the neural activity of the antennal lobe. (A)** Real image of the brain demonstrating the regions from which measurements of the neural activity were made in the antennal nerve and the macroglomerular complex. Scale bar = 200 μm. AN, antennal nerve; AL, antennal lobe; Gs, ordinary glomeruli; MGC, macroglomerular complex; Oe, esophagus; OL, optic lobe; PC, protocerebrum; SOG, suboesophageal ganglion. **(B)** Serotonin had no effect on the optical response in the antennal nerve. Gray line, control response; black line, response following serotonin application. **(C)** The neural activity in the macroglomerular complex was enhanced greatly by serotonin. **(D)** The neural activity in the antennal lobe in response to electrical stimulation of the antennal nerve was greater and longer lasting following the application of serotonin. The response was normalized by the signal from the antennal nerve. Normalized values of control (upper), serotonin application (middle), and the serotonin effect (bottom). (modified from Hill et al., 2003).

The neuronal mechanisms of increased sensitivity have been investigated in several moth species. In *B. mori*, the effects of serotonin on the AL have been investigated using voltage-sensitive dye imaging. Voltage-sensitive dyes change their fluorescence depending on the membrane voltage, allowing the gross activity level of the AL to be monitored. Most ORNs are pheromone-sensitive, and so it is possible to examine pheromone processing using optical recordings. When serotonin is applied, the response of toroid (which processes bombykol) is selectively enhanced (Hill et al., 2003) (Figure 8). In addition, the activity of several glomeruli in the OG is enhanced. In the related species *M. sexta*, serotonin changes the membrane properties and excitability of AL PNs (Mercer et al., 1995, 1996; Kloppenburg et al., 1999).

**Figure 9:**
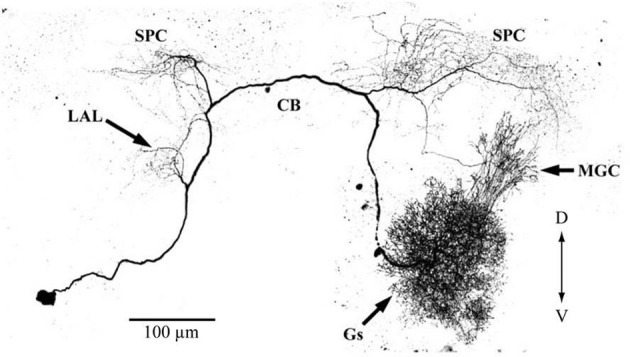
**Morphologies of putative serotonin feedback neurons.** The neuron has its soma in the posterior portion of the lateral cell cluster of one antennal lobe. The primary neurite projects through the ipsilateral antennal lobe, where it has a small number of fine branches in the posterior coarse neuropil. Branches are present in the ipsilateral lateral accessory lobe, and in both the ipsi- and contralateral parts of the protocerebrum, including the calyces of both mushroom bodies and the central body. The neuron shows immunoreactivity with anti-5HT antibody. CB, central body; D, dorsal; Gs, ordinary glomeruli; MGC, macroglomerular complex; LAL, lateral accessory lobe; SPC, superior protocerebrum; V, ventral. (modified from Hill et al., 2002).

An important question is what is the source of serotonin in the AL. Immunocytochemistry using anti-serotonin antibodies has revealed only a pair of serotonin-secreting neurons in the AL. These have been characterized using intracellular recording methods (Hill et al., 2002) (Figure 9), and have been suggested to be neuro-secretory structures based on the widths of their action potentials. Similar neurons have been described in other moths (Kent et al., 1987; Sun et al., 1993; Zhao and Berg, 2009) and in other insect taxa (Dacks et al., 2006), suggesting that they are homologous neurons with preserved functional and morphological features across species. These neurons have smooth processes in the ipsilateral LAL and ipsilateral and contralateral superior protocerebrum, and blebby processes in the contralateral LAL and contralateral AL, and are thought to form putative feedback neurons from the protocerebrum to the AL. This serotonin-like immunoreactivity has been observed in various brain regions, including the calyx of the MB, the LAL, and others. Therefore, there are many candidate circuits that translate the increased brain serotonin levels into increased pheromone sensitivity.

## Closing remarks

In more than half a century since the first identification of sex pheromones from female silkmoths (Butenandt et al., 1959), the sex pheromone reception and processing systems in male moths have played central roles in insect olfactory research. Accordingly, the silkmoth sex pheromone system is one of the most well understood olfactory systems. The fundamental molecular mechanisms of pheromone detection at the periphery are clear; the pheromone signals detected by the ORNs of male moths are coded by a labeled line for the initiation of pheromone-source searching behavior in the brain. The main pheromone-processing pathway is also well understood from the AL to the output motor neurons. Despite this progress, there remain important unanswered questions. For example, the signal transduction mechanisms for pheromone detection and the molecular determinants of pheromone specificity remain controversial. To fully understand the mechanisms of pheromone reception, it is important to not only examine the functional details of each molecular component, but also to unravel how all components interact in the context of the *in vivo* molecular network under different physiological conditions. The silkmoth has great potential to contribute to this understanding because methodologies for *in vivo* gene analysis such as the use of transgenes and gene targeting can be applied (Tamura et al., 2000; Imamura et al., 2003; Sajwan et al., 2013). When processing in the brain is considered, although it is clear that bombykol signals alone are sufficient to elicit pheromone-source searching behavior, this behavior is modified by the presence of other odorants, as well as the internal state of moths. To better understand pheromone processing, it is important to appreciate how pheromone-processing circuits are modulated via interactions with other neural circuits.

### Conflict of interest statement

The authors declare that the research was conducted in the absence of any commercial or financial relationships that could be construed as a potential conflict of interest.
